# Disturbances in the murine hepatic circadian clock in alcohol-induced hepatic steatosis

**DOI:** 10.1038/srep03725

**Published:** 2014-01-16

**Authors:** Peng Zhou, Ruth A. Ross, Cameron M. Pywell, Suthat Liangpunsakul, Giles E. Duffield

**Affiliations:** 1 Department of Biological Sciences and Eck Institute for Global Health, University of Notre Dame, Notre Dame, IN 46556; 2 Division of Gastroenterology and Hepatology, Department of Medicine, Indiana University School of Medicine, Indianapolis, IN 46202; 3 Roudebush Veterans Administration Medical Center, Indianapolis, IN 46202

## Abstract

To investigate the role of the circadian clock in the development of alcohol-induced fatty liver disease we examined livers of mice chronically alcohol-fed over 4-weeks that resulted in steatosis. Here we show time-of-day specific changes in expression of clock genes and clock-controlled genes, including those associated with lipid and bile acid regulation. Such changes were not observed following a 1-week alcohol treatment with no hepatic lipid accumulation. Real-time bioluminescence reporting of PERIOD2 protein expression suggests that these changes occur independently of the suprachiasmatic nucleus pacemaker. Further, we find profound time-of-day specific changes to the rhythmic synthesis/accumulation of triglycerides, cholesterol and bile acid, and the NAD/NADH ratio, processes that are under clock control. These results highlight not only that the circadian timekeeping system is disturbed in the alcohol-induced hepatic steatosis state, but also that the effects of alcohol upon the clock itself may actually contribute to the development of hepatic steatosis.

A cell-autonomous circadian clock controls local hepatic physiology by driving rhythmic gene and protein expression, and these in turn impact time-of-day specific regulation of many processes including lipid and glucose metabolism, and bile acid (BA) synthesis^1^. The molecular clock is composed of a self-sustainable pacemaker that generates 24-h rhythmicity, an input pathway that allows the clock to be reset by environment temporal cues, and output mechanisms that regulate molecular pathways and ultimately lead to rhythms in biochemistry and physiology. Though it is known that the master circadian oscillator resides in the hypothalamic suprachiasmatic nucleus (SCN), peripheral tissues also contain self-sustaining clocks^2^,^3^. At the molecular level, the circadian clock is composed of a series of autoregulatory transcriptional translational feedback loops (TTFLs): a negative loop consisting of *period* (*per*) and *cryptochrome* (*cry*) genes; a positive loop comprising the heterodimerization of bHLH-PAS proteins BMAL1 and CLOCK or NPAS2, that act through E-box regulatory elements in their target genes; and an interconnecting loop comprising ROR*α/β/γ* and REV-ERB*α*/*β*, which activate and inhibit transcription, respectively, by acting upon ROR elements in Bmal1 and clock/Npas2 gene promoters^2^.

Clock output is a critical aspect of the circadian system, connecting the core molecular oscillator with the rhythmic regulation of cellular effectors (e.g. enzymes, hormones, ion channels), and ultimately generating 24-h rhythms of physiology. This is achieved primarily through rhythmic regulation of clock-controlled genes (CCGs). A proportion of CCGs can be described ‘primary output genes’ (POGs), which are driven on consensus sites by clock proteins and are themselves transcriptional regulators^2^,^4^. These impart time-of-day-specific regulation on downstream CCGs, such as cellular effectors. POGs include *E4bp4* (also known as *Nuclear factor, interleukin 3 regulated* [*NIFL3*]), *D-box binding protein* (*Dbp*), *thyrotrophic embryonic factor* (*Tef*), and *hairy and enhancer of split 6* (*hes6*)^2^,^4^,^5^. Other rhythmically expressed transcriptional and posttranscriptional regulators, such as *Inhibitor of DNA binding* genes (*Id1*-*Id4*), nocturnin and certain microRNAs such as *miR122a*, also have the potential to serve in this time-of-day specific regulatory manner^2^,^6^,^7^,^8^. In fact, rhythmic expression can be controlled by multiple transcription factors acting in concert: *Cytochrome P450, family 7, subfamily A, polypeptide 1* (*Cyp7a1*), which encodes a rate-limiting enzyme controlling synthesis of bile from cholesterol, is regulated by DBP, REV-ERB*α*/*β* and E4BP4^9^,^10^,^11^. This collaboration allows for the amplitude, phase and waveform of CCG rhythmic profiles to be finely controlled^10^,^11^.

Hepatic steatosis, the accumulation of triglyceride [TG] droplets in the hepatocytes, is the earliest response of the liver to excessive alcohol use^12^. Alcohol metabolism reduces the nicotinamide adenine dinucleotide/reduced nicotinamide adenine dinucleotide (NAD/NADH) ratio resulting in interrupted mitochondrial fatty acid (FA) β-oxidation and thus hepatic steatosis^12^. Alcohol exposure, directly or indirectly, regulates transcription factors, such as sterol regulatory element-binding protein 1c (SREBP-1c) and peroxisome proliferator-activated receptor α (PPARα), leading to lipogenesis and inhibition of FA oxidation, respectively^12^. Genes under the control of these transcription factors are disturbed by alcohol consumption, including *acetyl Co-A carboxylase* (*ACC*) and *fatty acid synthase* (*Fas*) in the FA synthesis pathway, and *3-hydroxy-3-methylglutaryl-CoA reductase* (*hmgcr*) in cholesterol synthesis^12^. Alcohol consumption also interferes with BA synthesis. Plasma bile is elevated in patients with alcoholic hepatitis^13^, and rats chronically fed a high alcohol diet have increased bile secretion^14^, possibly due to elevated hepatic CYP7A1 expression^15^.

FA, cholesterol and BA synthesis fluctuate over the diurnal cycle with a circadian periodicity^16^,^17^,^18^,^19^, as do genes associated with their regulation^1^,^9^,^10^,^19^,^20^. The circadian clock not only drives rhythms in metabolic parameters but can also be modulated by them in the form of interlocking relationships^1^. For example, an altered redox state, which occurs with alcohol drinking^12^,^21^, plays an important role in circadian clock function in which NADH promotes binding of heterodimeric clock transcription factor complexes to DNA^1^,^22^,^23^. We therefore hypothesized that alcohol consumption would result in time-of-day specific changes in lipogenic and cholesterogenic responses and BA synthesis/accumulation. Here we show that chronic alcohol feeding [CAF] in mice interferes with the normal operation of the hepatic circadian clock, leading to the loss in temporal coordination of liver associated metabolic function at both the gene expression and physiological levels.

## Results

### Effect of short-term and chronic alcohol feeding on hepatic clock gene expression and time-of-day specific lipid panel profiles

**Figure 1:**
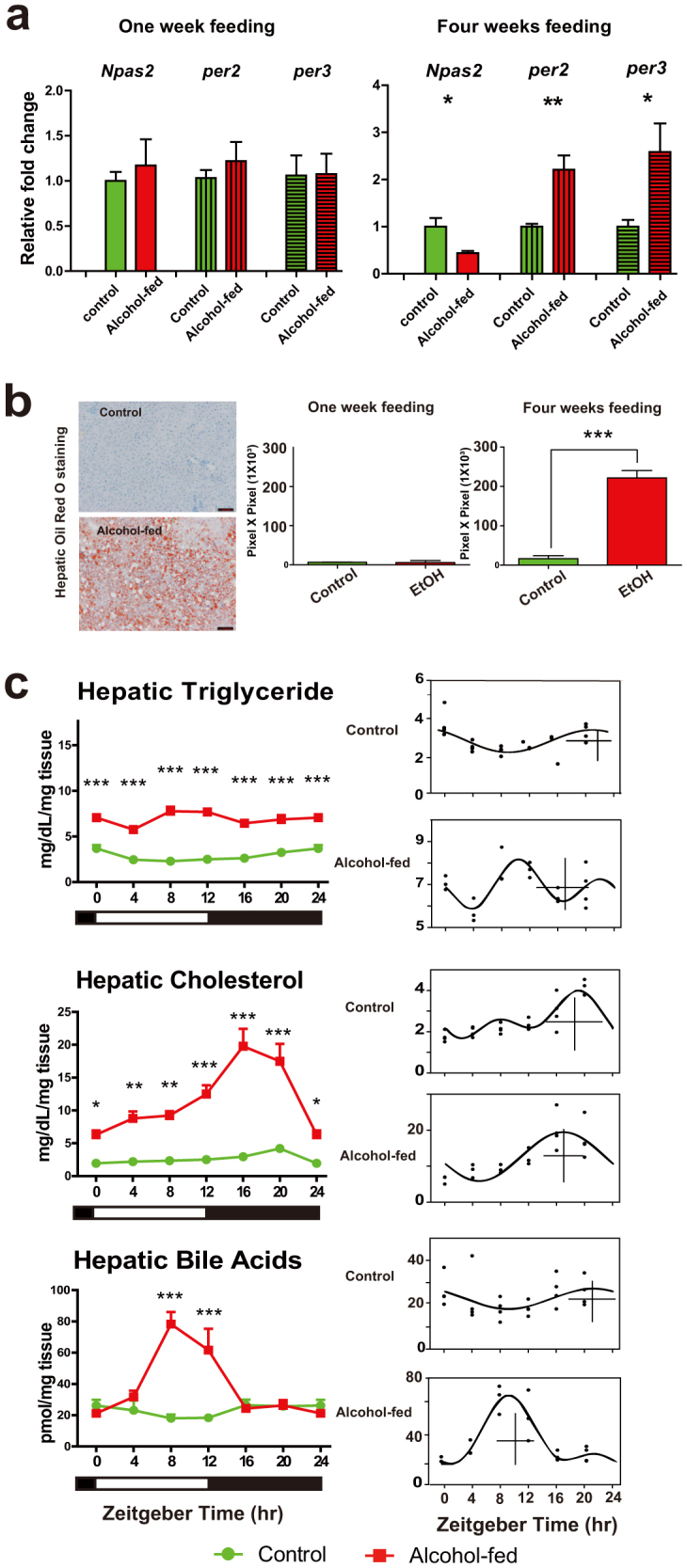
Effect of alcohol feeding on mouse liver. (a). qRT-PCR analysis of circadian clock gene expression from control and alcohol-fed mice. One-week (left) and 4-week feeding (right). Values are mean ± S.E.M., and where control group expression = 1.0. *t* tests (*p < 0.05, **p < 0.01). (b) Representative Oil Red O stained liver sections from control and alcohol-fed groups detecting neutral lipids (Left). Scale bars, 200 μm. Quantification values are mean ± S.E.M, n = 6 mice per group (right). *t* tests (***p < 0.001). (c) (Left) Hepatic lipid profiles (TG, CHOL, and TBA) in control (green line) and alcohol-fed (red) mice (n = 3–6 mice per time point) across 24-h cycle. Data from ZT0/24 are double-plotted. Two-way ANOVA performed followed by Tukey's post-hoc *t* tests (*p < 0.05, **p < 0.01, ***p < 0.001). (Right) Corresponding CircWave analysis of TG, CHOL and TBA in control and alcohol-fed mice. In the graph, each dot represents the level of compound for each animal in time-specific groups, the curve represents the best-fit fourier curve, the CG is represented by the vertical bar, and the mean of the entire data set is represented by the cross.

The effect of alcohol on circadian gene expression was studied in both short-term and CAF models. Mice were fed with a Lieber-DeCarli diet for 1- week or 4-weeks^24^. Hepatic expression of circadian clock genes *per2*, *per3* and *Npas*2 were measured by real-time quantitative reverse transcriptase-polymerase chain reaction (qRT-PCR) at Zeitgeber Time [ZT] 2.5 (ZT0 = lights on, ZT12 = lights off). There were no observed changes in their expression between treatment groups after 1-week of feeding (*t* tests, n.s.; Fig. 1a). However, in the 4-week protocol, *Npas*2 expression was decreased by ~60% compared to controls (Fig. 1a), whereas *per2* and *per3* expression was increased by 2.2-fold and 2.5-fold, respectively. Interestingly, the alterations in these clock genes coincide with the development of steatosis, which occurred only with CAF (Fig. 1b). Next, we characterized the effects of CAF on time-of-day specific lipid and BA profiles. The levels of intrahepatic TG, total cholesterol [CHOL] and total bile acid [TBA] were measured across the 24-h diurnal cycle (Fig. 1c). TG and CHOL were elevated at all time points in chronic alcohol-fed mice (ANOVA, TG: Time (Ti), *p* < 0.01; Treatment (Tr), *p* < 0.001; Interaction (I), *p* < 0.01; CHOL: Ti, *p* < 0.001; Tr, *p* < 0.001; I, *p* = 0.062). When we considered the individual data plots for each compound and treatment group generated from the CircWave analysis (Fig. 1c), we found a distinct phase shift in the rhythm of TG and CHOL abundance in the alcohol-fed groups. Notably, the center of gravity (CG) in the alcohol-fed group TG and CHOL profiles were phase advanced by 5-h and 1-h, respectively.

CAF also modified the rhythm of TBA synthesis/accumulation. TBA in alcohol-fed mice was increased in the mid- to late-day phase (ZT8, ZT12; ANOVA: Ti, *p* < 0.05; Tr, *p* < 0.001; I, *p* < 0.001). Moreover, the CG of the cosinor analysis in alcohol-fed mice was 12-h out of phase with that of the corresponding profile for control animals (Fig. 1c).

### Effect of CAF on hepatic expression of clock genes and CCGs

**Figure 2:**
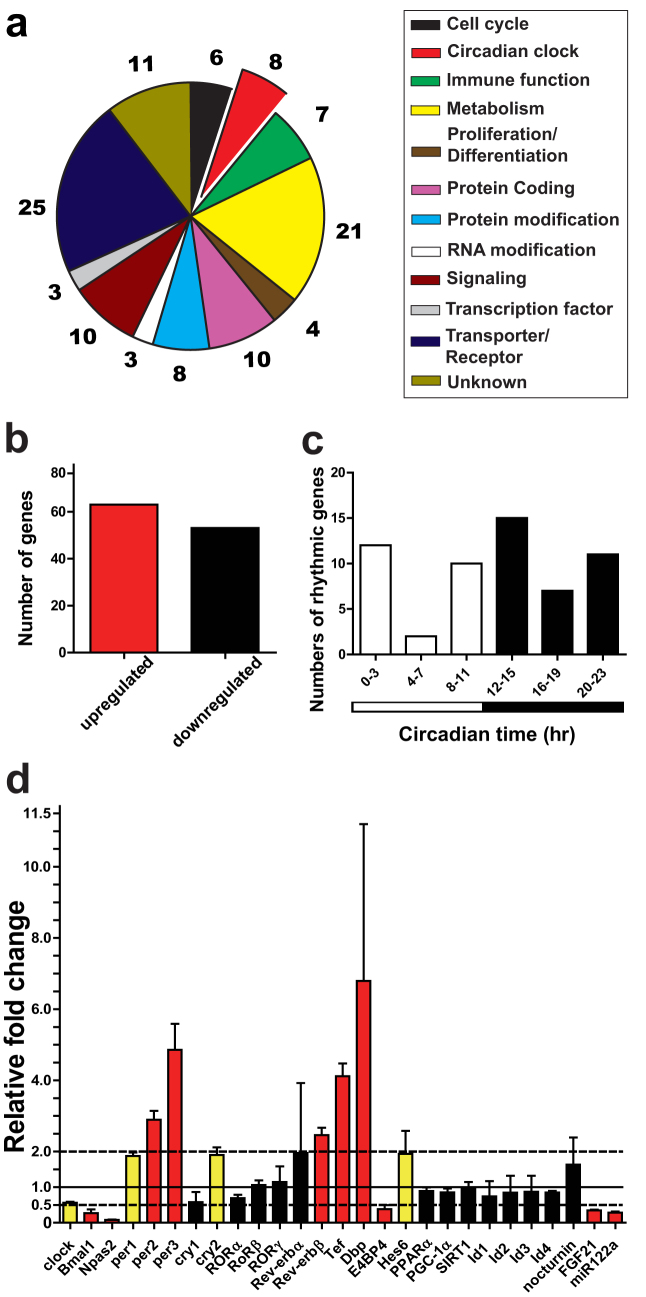
Microarray analysis of liver from alcohol-fed mice. (a) Genes involved in multiple cellular processes were identified as differentially expressed in alcohol-fed mice liver. Genes shown were scored as differentially expressed by ≥ 2-fold change. (b) 63 and 53 genes identified as differentially expressed were up- and down-regulated, respectively. (c) Distribution of differentially expressed CCGs according to known peak phase (determined from published database^51^,^52^). (d) Fold-change of selected differentially expressed genes from microarray experiment. Data represents mean ± SD relative to mean of control gene expression ( = 1.0). Red bar, p < 0.05 and ≥ 2-fold change; yellow bar, p < 0.05 and ≥ 1.5 to < 2-fold change; and black bar, p > 0.05.

To fully examine the effect of CAF on hepatic clock and CCG regulation, we performed a microarray analysis in murine liver collected at the same time of the day (ZT2.5). A total of 488 genes were differentially expressed between alcohol-fed and control mice (Fig. 2, Supplementary Fig. S1, Supplementary Datafile 1; *t* test, *p* < *0.05*, fold-change ≥ 1.5). Using a more stringent ≥ 2-fold expression change cut-off for differentially expressed genes, 116 genes were identified; 63 up- and 53 down-regulated, respectively (Fig. 2b, Supplementary Datafile 1). Of these were 8 canonical clock genes or POGs: *Bmal1, Npas2, per2, per3, Rev-erbβ, Dbp, Tef* and *E4bp4* (Fig. 2a). Twenty-one genes involved in metabolic processes represented the second largest functional group (Fig. 2a). Of importance, we found that 49% of differentially expressed genes were identified as CCGs (Supplementary Datafile 1). Twenty-five and 37 CCGs were found to peak during the day (Circadian time [CT] 0-CT11) and at night (CT12-CT23), respectively (Fig. 2c). When using > 1.5 and < 2 fold-change as a filter, 166 and 206 genes were found to be up- and down-regulated, respectively (Supplementary Fig. S1a, Supplementary Datafile 1), including three clock genes: *clock, per1* and *cry2*. Of these differentially expressed genes, 39% were identified as CCGs (e.g. *insulin induced gene 2* [*Insig2*] was down-regulated by 1.97-fold), and with more peak phases occurring at night than day (84 versus 65 genes; Supplementary Fig. S1b). After plotting all identified clock genes and CCGs against their respective circadian time of peak phase, the up-regulated genes have peak phases clustered from ZT8 to ZT16, while down-regulated genes peak from ZT20 to ZT4 (Supplementary Fig. S1c).

The core components of the clock, POGs and other important modulators of the clock, several of which were found differentially expressed in alcohol-fed mice, are shown in Fig. 2d. Consistent with our qRT-PCR results of CAF treatment examined at ZT2.5 (Fig. 1a), our microarray analysis revealed the up-regulation of *per2* and *per3* (by 2.9- and 4.9-fold) and down-regulation of *Npas2* (by 13-fold). Other clock genes, POGs and other important CCGs were up-regulated by alcohol feeding, including *per1* (1.9-fold), *cry2* (2-fold), *Rev-erbβ* (2-fold), *Dbp* (7-fold), *Tef* (4-fold), and *hes6* (2-fold). In contrast, clock genes involved the positive feedback loop of TTFL, *Bmal1* and *clock*, and the POG *E4bp4*, were down-regulated by 3.7-, 1.8-, 2.6-fold, respectively. The metabolic hormone gene *fibroblast growth factor 21* (*fgf21*) (2.9-fold) and the microRNA *miR122a* (3.5-fold), also recently implicated in contributing to the output network of the circadian clock^8^,^25^,^26^,^27^, were down-regulated.

### Differential expression of the core components of circadian clock, CCGs, and genes associated with lipid metabolism and BA synthesis

To understand the full extent of circadian regulation and rhythmicity of hepatic genes in alcohol-fed mice identified in our microarray analysis as differentially expressed in the liver of alcohol-fed mice, we next examined the expression of specific genes at 4-h intervals across the 24-h diurnal cycle.

#### Effect of CAF on core components of circadian clock

**Figure 3:**
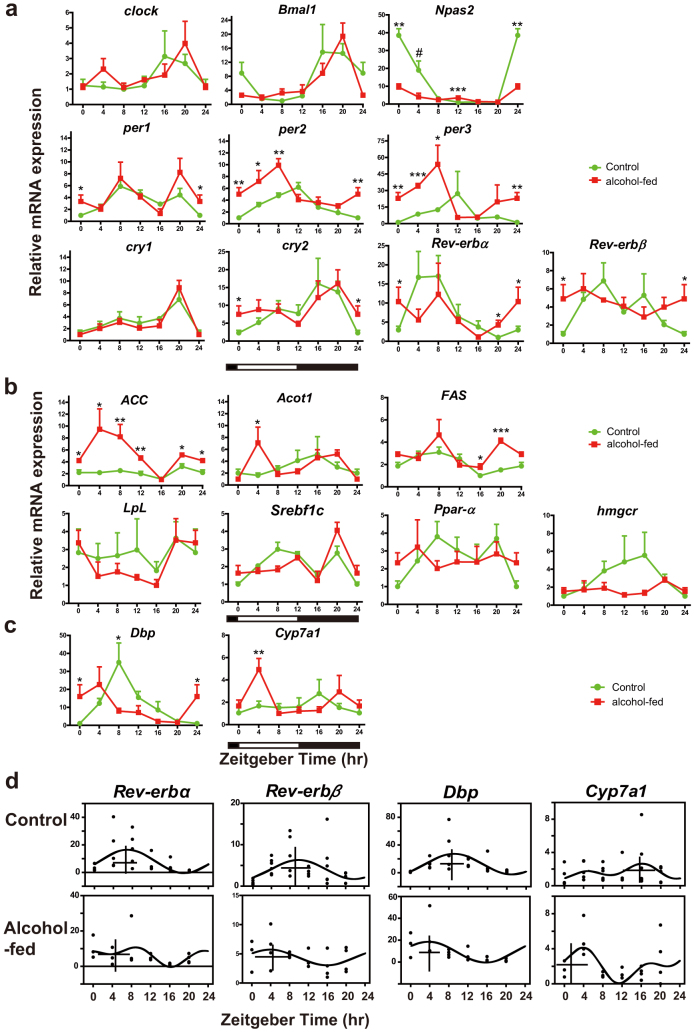
24-h analysis of clock gene, CCG and metabolic gene expression reveals time-of-day specific differences between alcohol-fed and control mice. (a) Clock gene expression in mouse liver. (b) Metabolism gene distribution in mouse liver. (c) *Dbp* and *Cyp7a1* gene expression. (d) CircWave analysis of *Rev-erbα*, *Rev-erbβ*, *Dbp* and *Cyp7a1* expression for control and alcohol-fed groups, showing CG. Values are mean ± S.E.M. expression relative to the lowest of time-specific mean value in entire series ( = 1.0; n = 3–6 mice per time point). Two-tailed Student's *t* tests (*p < 0.05, **p < 0.01, ***p < 0.001). For *Npas2* at ZT4, a one-tailed test was performed, since it was identified as up-regulated in the microarray analysis (#, p < 0.05). Data from ZT0/24 are double-plotted.

We found no change in the levels of *clock* and *Bmal1 a*cross the 24-h day between treatment groups (Fig. 3a). However, expression of another component of the positive limb of the TTFL, *Npas2*, was reduced by ~4-fold in the alcohol-fed group, notably at ZT0 and ZT4, and elevated at ZT12. For negative elements, *per1* was found elevated at ZT0 (Fig. 3a), consistent with the microarray analysis (Fig. 2d). More pronounced was *per2* and *per3* expression, which was elevated by 2-fold from ZT0-ZT8 in alcohol-fed mice (Fig. 3a). CircWave analysis revealed consistent results showing an advancing shift in the phase of the rhythms (*per2*: control CG, ZT10.2, alcohol-fed CG, ZT6.5; *per3*: control CG, ZT10.6, alcohol-fed CG, ZT5.3; Supplementary Fig. S2). No difference was observed for *cry*1, but for *cry*2 there was elevated expression at ZT0, also consistent with the microarray analysis (Fig. 2d, Fig. 3a), and a corresponding shift in the phase of the rhythm (Supplementary Fig. S2). For the interlocking TTFL, expression of both *Rev-erbα* and *Rev-erbβ* was elevated at ZT0, and *Rev-erbα* additionally at ZT20 (Fig. 2d, Fig. 3a), and with a phase advance in the CG of each rhythm (Fig. 2d, Supplementary Fig. S2).

There was a positive correlation between microarray experiment fold-change and qRT-PCR expression level data for ZT0 (*p* < 0.001; Fig. S1d). We next analyzed the peak to trough amplitudes of the clock gene rhythms and observed apparent differences (Table 1). Moreover, the basal levels of *per2* and *Rev-erbβ* in the alcohol-fed group were elevated (Fig. 3a). The amplitudes of *Bmal1, Npas2, per2, per3, cry2, Rev-erbα* and *Rev-erbβ* were lower in alcohol-fed group. No amplitude change was observed for *clock* or *per1*, and *cry1* had a larger amplitude.

#### Effect of alcohol feeding on CCGs associated with lipid metabolism and BA synthesis

We examined CCGs associated with fatty acid synthesis [*ACC*, *Fas*, *Srebpf-1c*], fatty acid oxidation [*pparα*, *acyl-coenzyme A thioesterase* (*Acot1*)], lipoprotein [*lipoprotein lipase* (*Lpl*)] and cholesterol metabolism [*hmgcr*] (Fig. 3b and Supplementary Fig. S2), and found several of these rhythmic genes had changes in their temporal profiles: *ACC* was elevated at almost all time points (ZT0-ZT12, ZT20); *Acot1* was elevated at ZT4; while *Fas* had an additional late peak at ZT16 and ZT20. The results were a change in the normal waveform of the rhythms, with the apparent occurrence of a second peak, resulting in bimodal temporal profiles. These changes were also reflected in the CircWave analysis in which either no cosinor could be fit for the alcohol group (*Acot1*, *Fas*), or resulted in a shift in the CG (*ACC*) (Supplemental Fig. S2). There was no significant difference in the expression levels of *Lpl, Srebpf-1c, pparα* or *hmgcr* between treatment groups.

Hepatic TBA synthesis is under clock control, involving coordinated expression of *Dbp, Rev-erbα/β* and *E4bp4*, in turn regulating the temporal expression of *Cyp7a1*^9^,^10^. We found both *Dbp* and *Cyp7a1* were up-regulated in the alcohol-fed group at ZT4 (Fig. 3c), and *E4bp4* was down-regulated (tested at ZT2.5 only; Fig. 2b). Of importance, there were shifts in the phases of *Rev-erbα*, *Rev-erbβ* and *Dbp* (Fig. 3c,d). When we considered the temporal profile of the gene target of these clock components, *Cyp7a1*, we also found its diurnal waveform was profoundly altered: peak expression occurred at different phases of cycle in a biphasic pattern, with a major peak at ZT4, and the CG of expression was anti-phasic, dramatically delayed by ~11-h (Fig. 3d).

### Bioluminescence analysis of hepatic and SCN PER2 protein expression

The circadian clock in peripheral tissues, such as liver, is entrained in part by the central light-entrainable clock in the SCN^2^,^3^. As CAF resulted in an up-regulation of hepatic *per2* and *per3* expression, we wished to determine whether such an effect of alcohol was mediated in part through an effect on the SCN. We studied the brain and liver derived from PERIOD2::LUCIFERASE (PER2::LUC) mice fed with control or CAF diets. This approach allows for examination of tissue-specific circadian responses independent of extrinsic systemic factors.

**Figure 4:**
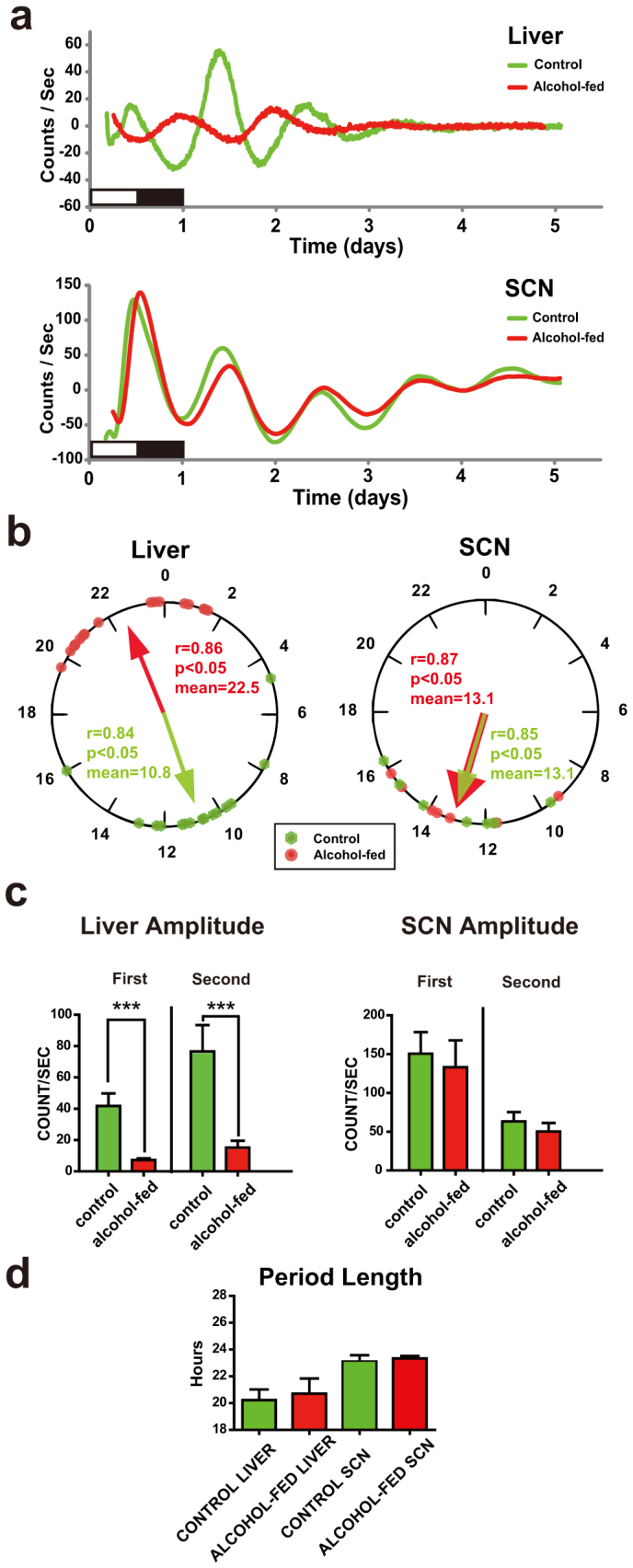
Bioluminescence recording of PER2 rhythms of alcohol-fed mice. (a) Circadian expression of PER2::LUC protein from a representative culture of liver and SCN explants from control (green line) and alcohol-fed (red line) mice. Data plotted as baseline subtracted. (b) Peak phase distribution of liver explants from control (n = 14 explants) and alcohol-fed mice (n = 17 explants) (left), and distributions of SCN explants (right). The mean phases of the each group are indicated by the arrows within the 24-h circle; arrow length represents the degree of synchrony of each group (r). In liver and SCN, phase distributions of each treatment group are different from uniform (Rayleigh test, p < 0.05); and specifically in the liver, distributions are different between the alcohol-fed and control groups (Mardia-Watson-Wheeler test, p < 0.001). (c) Amplitude of PER2::LUC rhythm in liver (left) and SCN (right) explants for the first and second cycles in culture. Values are mean ± S.E.M. of counts/second. ***p < 0.001. (d) Period length of PER2::LUC rhythm in liver and SCN explants. Student's *t* tests (n.s.).

Representative baseline subtracted traces for PER2 protein driven bioluminescence in the SCN and liver explants from control and alcohol-fed mice are shown in Fig. 4a. Using the peak of circadian oscillation in luminescence within the first 24-h of isolation, the phase distribution map of liver and SCN explants from control and alcohol-fed mice was constructed (Fig. 4b). There was no difference in the PER2::LUC expression of SCN derived from control and alcohol-fed mice. Conversely, for the liver the mean peak of PER2::LUC expression between the alcohol-fed and control mice was found to be in anti-phase, occurring in the alcohol-fed group at ZT22.5 (Fig. 4b). The alcohol-fed liver also exhibited a lower amplitude rhythm (Fig. 4c), a finding that was consistent with the *per2* gene expression (Fig. 3a; Table 1). No amplitude change was detected in SCN explants, and no difference was observed between treatments on period length in either tissue (Fig. 4d).

### Altered diurnal profile of NAD/NADH ratio in alcohol-fed mice

**Figure 5:**
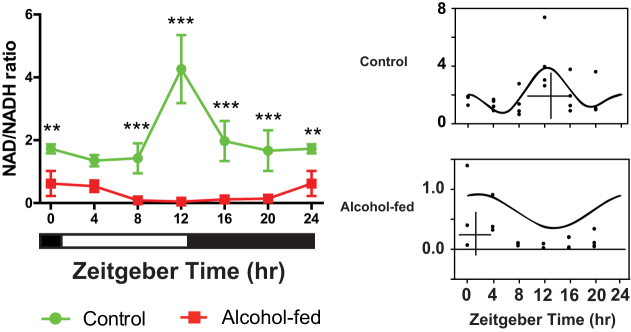
Alcohol treatment perturbs the normal diurnal rhythm of liver NAD/NADH ratio. 24-h rhythm of hepatic NAD/NADH for control and alcohol-fed mice (left); and CircWave analysis reveals an antiphasic relationship between treatment groups (right). Two-way ANOVA with Tukey's post-hoc tests, **p < 0.01, ***p < 0.001.

Previous work has revealed the regulation of circadian clock is partly mediated by changes in the NAD/NADH ratio^23^,^28^,^29^,^30^. We found that the NAD/NADH ratio was considerably lower following CAF (Fig. 5). The ratio was dramatically decreased at all times of the 24-h day (Ti, *p* = 0.1; Tr, *p* < 0.001; I, *p* < 0.01), but especially at ZT8–ZT20. Furthermore, there was a change in the phase of the diurnal rhythm, as shown by the CircWave CG, and where the alcohol-fed group (ZT1.1) was in anti-phase to the control group (ZT13.0).

## Discussion

In our study, we provide the novel finding that chronic alcohol feeding (CAF) results in the disturbance of the hepatic circadian clock and time-of-day specific regulation of lipid and BA synthesis/accumulation. Furthermore, these changes appear to be related to the direct effect of alcohol on the liver and not acting though the SCN pacemaker.

After 4-weeks, but not 1-week of alcohol feeding, gene expression of the components of the negative limb of the TTFL clock mechanism (*per2*, *per3*) were up-regulated, and positive elements (*Npas2*) down-regulated. This provides compelling evidence for an alteration of the hepatic clock produced by a chronic, instead of acute effect of alcohol. These alterations are consistent with changes in hepatic lipid accumulation, where no accumulation is observed after 1-week of feeding, but it is profoundly elevated after 4-weeks. Lipid accumulation observations are consistent with prior reports^24^. Previous studies demonstrate that intrahepatic lipid and BA syntheses are under circadian regulation and express diurnal rhythmicity under normal physiological conditions^16^,^17^,^18^,^19^,^31^. In our study, both the absolute levels and phase of rhythm of lipid and TBA were altered following CAF.

Microarray analysis revealed many components of the hepatic circadian system to be altered following CAF, highlighted by differential expression of clock genes (*clock, Bmal1, Npas2, per1*, *per2*, *per3*, *cry2*, *Rev-erbβ*), POGs (e.g. *Dbp*, *E4bp4*) and CCGs. In previous studies, only 3–9% of genes appear to be controlled by the clock in the liver^1^,^2^,^4^,^20^. In our study, ~40% of the genes found to be differentially expressed following CAF were identified as CCGs, this being far more than what would be predicted by chance. Almost all up-regulated CCGs had peak phases that clustered around CT8–CT16, and down-regulated CCGs at ~CT20-CT4. Thus, these changes in expression are occurring in a highly coordinated fashion, and suggest that the effect of chronic alcohol and/or steatosis is operating upon these genes via the circadian clock. Taken collectively, our results reveal that CAF and associated liver steatosis profoundly alter hepatic expression of canonical clock genes and CCGs.

The 24-h analysis revealed a diverse response of clock genes, POGs and CCGs to CAF, from an increased amplitude and phase shifts (*per2, per3, Rev-erbα, Dbp*, *Cyp7a1, ACC*), to damped expression (*Npas2*), and double peaks within the 24-h profile (i.e. bimodal pattern; *ACC*, *Acot1*, *Fas, Cyp7a1*). These results clearly reveal an influence of CAF on gene expression that follows a highly time-of-day specific pattern.

As we had observed phase shifts and baseline level changes in *per2* expression, we characterized the corresponding temporal profile of PER2 protein by examining PER2::LUC activity. The bioluminescence experiments suggest that the SCN clock was unaffected by CAF, highlighting that CAF does not have a broad effect on the circadian system but is tissue-specific. Consistent with mRNA analysis, the hepatic PER2 rhythm of alcohol-treated mice was phase-shifted, albeit protein was in anti-phase and mRNA ~6-h advanced. This discrepancy could be explained by an absence of competing time-giving signals exogenous to the liver, derived indirectly from the SCN pacemaker (e.g. temperature, glucocorticoids and time-specific feeding signals)^2^,^3^,^32^ or an absence of alcohol in the explant medium. This suggests that either the liver PER2 rhythm *in vivo* is anti-phasic, or that isolation of liver *ex vivo* from competing extrinsic signals released it to express own tissue-autonomous phase. The PER2::LUC rhythm amplitude was smaller in the alcohol-fed mice, a finding also consistent with the corresponding *per2* profile. Taken together, we conclude that the CAF exerts a direct effect on the hepatic clock, independently from SCN regulation.

**Figure 6:**
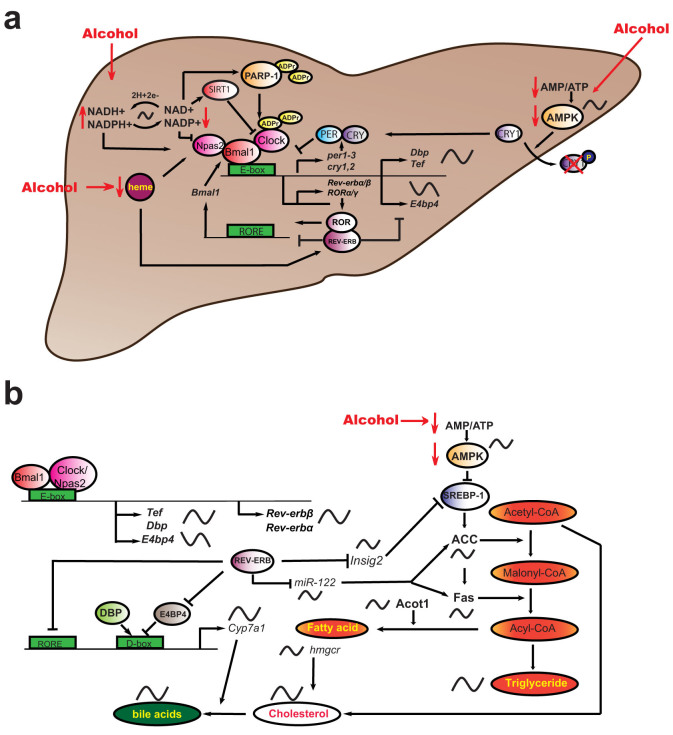
Model for effect of alcohol on hepatic circadian and metabolic systems. (a) Alcohol affects the circadian system through three main pathways: 1) alteration of intracellular redox state via NAD/NADH, thereby influencing activity of positive TTFL components. The CAF effect is partly mediated through altering the redox state, and leading to a decrease in the NAD/NADH ratio and in an anti-phase shift in its diurnal profile. The clock is regulated by the NAD/NADH ratio, whereby NADH can enhance DNA-binding activity of NPAS2:BMAL1 heterodimers, and NAD^+^ inhibits DNA-binding activity^23^. NAD^+^ regulates the activity of the deacetylase SIRTUIN1, thereby altering CLOCK:BMAL1-dependent transcription^28^,^29^. Intersecting feedback loops also result in NAD^+^ being under clock regulation^28^,^29^. NAD^+^ also acts as an adenosine diphosphate (ADP) ribose donor for poly-ADP ribose-polymerase-1 (PARP-1), which can bind to the CLOCK:BMAL1 heterodimer and poly (ADP ribosyl)ate CLOCK^30^. Hence, low NAD/NADH ratio is predicted to cause an increase in CLOCK:BMAL1 on E-box binding and increase transcriptional activity of target clock genes (*per2, per3*, *cry2*, *Rev-erbα*/*β*) and CCGs (*Dbp, Tef, Hes6*); 2) reducing heme levels and thus affecting the transcriptional activity of clock components NPAS2 and REV-ERB*α*/*β*, which contain heme-binding motifs^34^,^35^. Hepatic heme concentration is reduced in rats chronically fed with alcohol^37^; and 3) cellular energetics via AMP/ATP ratio, in turn affecting CRY1 degradation. CAF has been shown to reduce the activity and phosphorylation of adenosine monophosphate activated protein (AMPK)^38^. AMPK is under rhythmic regulation and can phosphorylate CRY1, leading to CRY1 degradation^39^. (b) Downstream clock targets include enzymes involved in FA synthesis, which eventually results in altered regulation of TGs, cholesterol and BA synthesis. *Rev-erbα* participates in the transcriptional regulation of liver specific *miR-122*, which was differentially regulated in our study, and which regulates *Fas* and *ACC*^8^,^25^. Additionally, DBP and TEF contribute to the circadian transcription of *Acot1*^40^. REV-ERBα can also regulate circadian BA and lipid homeostasis via cyclic expression of *Insig2* and its subsequent action on SREBP activity^41^. CYP7A1 is the rate-limiting enzyme for synthesis of bile acids, and its expression is regulated by DBP and REV-ERB*α*/*β*. Rhythmically expressed components are indicated by sinusoidal lines.

In order to explain how alcohol regulates the hepatic clock we propose a model (Fig. 6a). In our study, NAD/NADH ratio following CAF was found to be considerably reduced. Part of this response is likely the result of increased cytosolic and mitochondrial NADH generated by alcohol and aldehyde-dehydrogenases^21^,^33^. Furthermore, we observed a dramatic anti-phase shift in NAD/NADH diurnal profile. Several studies have revealed that the circadian clock is directly regulated by the NAD/NADH ratio: NADH can enhance DNA-binding activity of NPAS2:BMAL1 heterodimers, whereas NAD^+^ inhibits DNA-binding activity^23^. NAD^+^ also regulates the activity of the deacetylase enzyme SIRTUIN1, thereby altering CLOCK:BMAL1-dependent transcription^28^,^29^. Intersecting feedback loops also result in NAD^+^ being under circadian clock regulation^28^,^29^. Furthermore, NAD^+^ acts as an ADP ribose donor for poly-ADP ribose-polymerase-1 (PARP-1), which can bind to the CLOCK: BMAL1 heterodimer and poly (ADP ribosyl)ate CLOCK^30^.

Based on the finding that CAF results in both a reduced NAD/NADH ratio and a shift in its diurnal profile, and known interactions of NAD^+^ with defined clock components and clock modulators terminating on positive TTFL components^22^,^23^,^28^,^29^,^30^, we propose that changes in hepatic NAD/NADH ratio likely constitutes a major modulator of the clock in CAF mice (Fig. 6a). Hence, low NAD/NADH ratio is predicted to cause an increase in CLOCK/NPAS2:BMAL1 on E-box binding and increase transcriptional activity of target clock genes and CCGs. In accordance with this hypothesis, we found that not only negative components of TTFL (*per2, per3*, *cry2*), but also interlocking loop components (*Rev-erbα*/*β*) and direct CLOCK/NPAS2:BMAL1 target POGs (*Dbp, Tef, Hes6*), were up-regulated in the liver of alcohol-fed mice during the morning-day phase (ZT0-12). Further, NPAS2 bears a heme-binding motif, and the activity of the BMAL1:NPAS2 transcriptional complex is regulated by heme^34^. Heme is also the ligand for REV-ERB*α*/*β*^35^,^36^. Previous reports have shown a reduction in hepatic heme concentration in rats chronically fed with alcohol^37^. In addition, chronic alcohol feeding has been shown to reduce the activity and phosphorylation of adenosine monophosphate activated protein (AMPK)^38^. AMPK is also under rhythmic regulation and can phosphorylate CRY1, leading to CRY1 degradation^39^ (Fig. 6a). This suggests potential roles for heme and AMPK in alcohol-induced hepatic clock alterations. Clearly there are multiple potential mechanisms through which the circadian clock TTFLs could be modulated by alcohol. The steady state of the hepatic clock in the CAF mice is likely to be the result of a balance between the negative and positive influences of these intersecting factors (NAD/NADH ratio^22^,^23^,^28^,^29^,^30^, heme^34^,^35^,^36^,^37^ and AMPK^38^,^39^) that are themselves rhythmically regulated, and acting upon specific elements of the molecular clock (Fig. 6a).

Our data also revealed phase-specific alterations in the rhythmic expression profiles of CCGs, presumably in response to the changes in the activity of the core clock. We predicted that changes in metabolic CCGs would lead to the observed metabolic changes in the liver of alcohol-treated mice (Fig. 6b). For instance, *Cyp7a1*, a major rate-limiting enzyme in BA synthesis, is under circadian control by DBP and REV-ERBα/*β* (acting on ROR elements, and by repressing expression of *E4bp4*)^9^,^10^. We found that the expression patterns of *Rev-erb*α/*β*, *Dbp* and *Cyp7a1* followed a temporal circadian sequence (and *E4bp4* was down-regulated when examined at ZT2.5). In alcohol-fed mice, the unique peak expression of *Dbp, Rev-erb*α and *Rev-erbβ* occurred at ZT0 followed by an increase in the level of *Cyp7a1* at ZT4. Moreover, the 4-h shift in the cosinor analysis determined CG of *Dbp* and *Rev-erb*α/*β* rhythms is associated with a 12-h phase shift in that of *Cyp7a1*. The alteration in the expression of genes involved in BA synthesis would explain the corresponding changes in the rhythmic peak of TBA concentration. We also observed a difference in the rhythmic profile of TG accumulation. In the control animals a single peak (ZT0) and trough (ZT8) were observed, but the alcohol-fed animals exhibited a bimodal profile, with a dominant peak ZT8 and lower peak at ZT0, and a CG phase advanced by 5-h. Such physiological alterations correlate with the changes in clock gene (*Rev-erbα*), POG (*Dbp, Tef*) and CCG expression (*miR-122*, *Insig2*, *ACC, Fas*, *Acot1*) (Fig. 6b). *Rev-erbα* participates in the transcriptional regulation of liver specific *miR-122*, which regulates *Fas* and *ACC*^8^,^25^, and also was differentially regulated in our study. In addition, DBP and TEF contribute to the circadian transcription of *Acot1*^40^. REV-ERBα can also regulate circadian BA and lipid homeostasis via cyclic expression of *Insig2* (differentially regulated in our study) and its subsequent action on SREBP activity^41^ (Fig. 6b). Together, the changes in the levels of intrahepatic lipid and BA in alcohol-fed mice can be explained by these alterations at the level of the hepatic molecular clock and subsequent disturbance in the expression of metabolic CCGs.

In conclusion, the hepatic clock is affected by chronic alcohol treatment and liver steatosis, as observed by changes at gene and protein expression; at the functional level, as observed by phase-specific changes in clock output; and at the physiological level, as observed by phase changes in lipid and TBA levels. However, the core clock still remains *functional*, albeit disturbed, as can be seen in the PER2::LUC rhythm *ex-vivo* and the patterns of clock gene and CCG expression *in vivo*. With all evidence considered, the following question arises: is CAF changing the circadian clock mechanism, which results in steatosis; or is steatosis, induced by CAF, changing the clock? It is also possible that CAF changes the clock and induces liver steatosis separately. A plausible explanation is that increased TG, CHOL and TBA levels are caused by CAF, induce steatosis, and then lead to an altered clock. Clock gene and CCG rhythms are also altered in high fat diet induced obesity^42^. However, they are not identical to what we observe here, making our scenario unique. Conversely, it is plausible that changes in the clock in response to CAF result in altered TG, CHOL and TBA synthesis/accumulation, in turn promoting steatosis. This notion is not impossible given that genetic manipulation of specific clock genes result in altered lipid metabolism, including obese phenotypes. For example, the *clock* mutant mouse is obese, the *Rev-erbα*−/− mouse has altered accumulation of liver CHOL and gallbladder BAs, and diet-induced obesity can be reversed by REV-ERB agonists^1^,^41^,^43^.

While our manuscript was in preparation, a similar study of CAF treated mice, Filiano *et al* (2013)^44^ reported differences in the diurnal expression of clock genes in liver, but only limited effects upon the SCN, a finding broadly consistent with the current study.

In summary, we have found that alcohol disturbs the hepatic circadian clock and CCGs, which might lead to the observed phenotype of hepatic steatosis and elevated intrahepatic BA. Since several genes involved in lipid and BA syntheses are under the regulation of clock, our study suggests that modulation of hepatic clock might be an alluring approach for treating alcohol-induced steatosis.

## Methods

### Animals and diets

C57BL/6 mice and PER2::LUCIFERASE mice (PER2::LUC; C57BL/6J background) were entrained to 12:12 light:dark (LD) cycle for ≥ 4 weeks and then fed with a Lieber-DeCarli liquid diet^24^. Mice were tested for effects of short-term and chronic alcohol exposure on hepatic clock at specific intervals across the 24-h LD cycle; and the effect of CAF on hepatic circadian clock function by real-time bioluminescence reporting of circadian protein expression, using established techniques^32^. Experiments were approved by the Indiana University Purdue University Indianapolis (IUPUI) and University of Notre Dame (UND) IACUCs, and performed in accordance with NIH Guidelines for the Care and Use of Laboratory Animals. Mice were male C57BL/6 from Jackson Laboratory (Bar harbor, ME) and PER2::LUC, generated from in-house breeding at UND^45^. Mice were 5 weeks old at time at start of entrainment, and 9 weeks at start of controlled feeding. The experimental design consisted of two dietary groups: 1) control diet (fat comprising 10% of total calories, with 6% from cocoa butter, and 4% from safflower oil; 72% of calories as carbohydrate); and 2) ethanol-containing diet [identical to the control diet, except with ethanol added to account for 27.5% of total calories and the caloric equivalent of carbohydrate (maltose-dextrin) removed]. Protein content of the diet was constant at 18% of calories, and each diet had identical mineral and vitamin content (Dyets Inc., Bethlehem, PA). Alcohol was introduced gradually into the diet during the first 5 days of feeding. Mice were fed with 9% alcohol (of the total calories) for 2 days, 18% for the next 3 days, and then finally 27.5% until the end of the experiment^24^,^46^.

#### The effects of short-term and chronic alcohol exposure on hepatic clock

To study the effects of short-term and chronic alcohol exposure on hepatic clock, mice (n = 5 per group) were housed individually in a room with controlled temperature (20–22°C) and humidity (55–65%). Animals were maintained on a 12:12 LD cycle and pair-fed with control and ethanol containing diets for 1-week and 4-weeks (not including the first 5 days of alcohol introduction). Food and separate water were available *ad libitum*. At the time of sacrifice, liver was harvested at Zeitgeber Time [ZT] 2.5 (ZT0 = lights on [morning], ZT12 = lights off [evening]).

#### The effect of chronic alcohol exposure on the full temporal profiles of hepatic clock

Mice were pair-fed with Lieber Dicarli diet for 4-weeks as outlined above. Mice were sacrificed and hepatic tissues were harvested at 4-h intervals (ZT0, 4, 8, 12, 16 and 20, n = 4–6 per group for each time point) representing all phases of the 24-h diel cycle. Tissues were immediately frozen with liquid nitrogen and stored at −80°C.

#### The effect of chronic alcohol feeding on hepatic circadian clock function by real-time luminescence reporting of circadian protein expression

PER2::LUC mice were pair-fed with control and ethanol containing diets for 4-weeks. Mice were sacrificed at ZT4 or ZT8. Explant cultures were prepared as described previously^32^. Briefly, brain and liver were quickly removed and placed in ice-cold Hanks Balanced Salt Solution (HBSS; Cellgro, Mediatech, Inc., VA). Coronal sections of the brain (300 μm thickness) were cut on a manual vibroslice (NVSL; World Precision Instruments, Inc., FL, USA). Brain regions were identified under a dissecting microscope and the SCN was isolated as a square tissue (~1.5 mm^2^), containing either a rostral or caudal aspect of the SCN. Liver was sectioned using the vibroslice at a thickness of 250 μm, and isolated in a square of ~1 mm^2^. Explants were cultured using an established method on a polypropylene mesh, which created an interface between culture medium and humidified air and explants were housed within sealed culture dishes^47^. SCN explants were cultured on culture plate inserts (Millicell, Millipore, MA, USA), and liver samples were placed on a mesh [Polypropylene Spectra/Mesh (210 μm opening, 308 μm thickness, 34% open area)], Spectrum laboratories, Inc., CA, USA). All tissue samples were placed in individual 35 mm translucent petri dishes with 1.2 ml of culture media composed of Dulbecco's Modified Eagle's Medium (Sigma-Aldrich), 19 mM D-glucose, 4.2 mM NaHCO3, 10 mM HEPES, 25 U/ml penicillin and streptomycin, 5% FBS and 100 mM luciferin (Luck-100, Gold Biotechnology, MO, USA), and tissue samples were sealed with vacuum grease and glass coverslips^47^. Tissues were placed in a light tight box at 36°C and bioluminescence was measured using a photomultiplier tube (LumiCycle, ActiMetrics, IL, USA) every 10 mins over 4 days.

### Real-time quantitative RT-PCR (qRT-PCR) analysis

RNA extraction was performed as described previously^6^,^48^,^49^. Total RNA was prepared from liver using an Absolutely RNA RT-PCR Miniprep kit (Stratagene, Cedar Creek, TX). Reverse transcription of 1 μg total RNA to cDNA was performed using a StrataScript qPCR cDNA synthesis kit (Stratagene). qRT-PCR was performed using a Stratagene MX 3005P thermal cycler (La Jolla, CA). SYBR green reagents were used and quantification was based on the generation of standard curves^7^. Gene expression was normalized relative to 18S ribosomal RNA, previously shown to be constitutively expressed over 24-h cycle^6^,^49^. Primers were purchased from Qiagen (Valencia, CA): *clock* (Cat No PPM24994A, Refseq accession No NM007715), *Bmal1* (Cat No PPM25679F, Refseq accession No NM007489), *Npas2* (Cat No PPM24791A, Refseq accession No NM008719), *per1* (Cat No PPM05041B, Refseq accession No NM011065), *per2* (Cat No PPM25497F, Refseq accession No NM011066), *per3* (Cat No PPM30973A, Refseq accession No NM011067), *cry1* (Cat No PPM26864A, Refseq accession No NM007771), *ACC* (Cat No PPM05109F, Refseq accession No NM133360), *Acot1* (Cat No PPM24730B, Refseq accession No NM012006), *Fas* (Cat No PPM03816F, Refseq accession No NM007988), *Lpl* (Cat No PPM04353F, Refseq accession No NM008509), *srebf-1c* (Cat No PPM05094A, Refseq accession No NM011480), *pparα* (Cat No PPM03307C, Refseq accession No NM011144) and 18s ribosomal RNA (Cat No PPM72041A, Refseq accession No NR003278). The primer sequences of other studied genes, purchased from Integrated DNA Technologies (San Diego, CA), were: *cry2*: F- 5′- GAT GTG TTC CCA AGG CTG TTC AAG -3′; R- 5′- GAG TTC TCA GTC ACC ACC TCC ACG -3′, *Rev-erbα*: F-5′- TGG CAT GGT GCT ACT GTG TAA GG -3′; R-5′- ATA TTC TGT TGG ATG CTC CGG CG -3′, *Rev-erbβ*: F-5′- GGA GTT CAT GCT TGT GAA GGC TGT -3′; R- 5′- CAG ACA CTT CTT AAA GCG GCA CTG -3′, *hmgcr*: F-5′- AGC TTG CCC GAA TTG TAT GTG -3′; R-5′- TCT GTT GTG AAC CAT GTG ACT TC -3′, *Dbp*: F-5′- AGG CAA GGA AAG TCC AGG TG -3′; R- 5′- TCT TGC GTC TCT CGA CCT CTT -3′, *Cyp7a1*: F- 5′- AGC AAC TAA ACA ACC TGC CAG TAC TA -3′; R- 5′- GTC CGG ATA TTC AAG GAT GCA -3′. No primer pairs generated quantifiable primer dimers.

### Histology

Liver sections (10 μm) were fixed in 10% formalin and processed for oil-red-O and hematoxylin-eosin staining.

### Hepatic TG, CHOL and TBA concentration assays

Twenty mg of liver powder was prepared under liquid nitrogen. Lipid extractions were performed using isopropanol, and hepatic TG and CHOL contents were measured using Wako L-type TG H and Cholesterol assay kits, respectively (Wako Diagnostics, Richmond, VA)^50^. Hepatic TBA measurements were performed as previously reported using the SekiSui Diagnostic kit (Exton, PA)^19^.

### Microarray analysis

Expression profiling was performed at the Microarray Core Facility, Dana Farber Cancer Institute (Boston, MA) using Mouse Gene 1.0 ST Arrays (Affymetrix, Santa Clara, CA). cRNA (200 ng/ul) was isolated from liver harvested at ZT2.5 from controls and alcohol-fed mice (4-weeks of treatment, n = 2 per group). Expression values were normalized (GC-RMA algorithm in GeneSpring GX11; Agilent Technologies, Santa Clara, CA) and Raw (.cel) files were imported using Partek Genomics Suite (Partek, St. Louis, MO). RMA background correction was performed and expression values were transformed to a Log_2_ scale. Genes differentially expressed between groups were identified by making pairwise comparisons using *t*-test analysis. For each comparison, gene networks and pathway analysis were performed on genes differentially expressed with p < 0.05 with Ingenuity pathway analysis (Ingenuity Systems, Redwood City, CA) software. CCGs were identified from Circa database of mouse liver sampled by microarrary as a 48-h circadian time course (http://bioinf.itmat.upenn.edu/circa)^51^,^52^ and where we defined CCGs as a JTK_CYCLE algorithm determined q < 0.1 value and a period length of 20–28-h^52^,^53^,^54^.

### Bioluminescence data analysis

Bioluminescence data from PER2::LUC mouse SCN and liver explants were de-trended by subtracting 24-h running average from the raw data. Explants expressing only one circadian peak were considered arrhythmic. A robust and sustained circadian rhythm of bioluminescence was found in the SCN, and ≥ 3 days of rhythms were observed for the liver. The peak of the circadian oscillation in culture was determined by measuring the highest point in the first complete cycle *in vitro*^32^,^45^. In control tissues, the mean peak of PER2::LUC expression occurred at ZT13 (for SCN), and ZT11 (for liver); which are consistent with previous studies^32^,^55^. Amplitude was calculated from the difference between the first peak and trough, and between the second peak and first trough. Period length of was determined by the mean time interval between each peak over 3 days.

### NAD and NADH assay

Hepatic NAD and NADH concentrations were assessed using the EnzyChrom NAD^+^/NADH Assay kit (ECND-100, BioAssay Systems, Hayward, CA).

### Statistical analysis

For qRT-PCR analysis, two-tailed Student's *t*-tests (Sigmaplot version 11; Systat Software, Inc., Chicago, IL, USA) were performed at individual time points to determine the statistical significance between alcohol-fed group and control groups. CircWave v1.4 software (www.huttlab.nl, www.euclock.org; an extension of the Cosinor analysis, courtesy of Dr. Roelof Hut) was used to analyze the rhythmicity of gene expression by fitting a Fourier-curve (one sine wave with up to two additional harmonics) to the data. *p*-values reported are the result of an F-test from software, user defined α = 0.5^56^. Graphs marked with ‡ represent data sets to which no curve could be fit. For hepatic biochemical analysis (TG, CHOL and TBA concentration) and NAD/NADH ratio, two way ANOVA was performed between alcohol-fed and control groups with Tukey's post-hoc tests. Where necessary, data were log or square root transformed to correct for non-normal distributions. Pearson correlation coefficient was used to test the relationship between time-specific expression values of clock genes between qRT-PCR and microarray analyses. The Rayleigh test was used for bioluminescence data analysis to determine whether two distributions of data were different from uniform; and the Mardia-Watson-Wheeler test was applied to assess for differences between the distributions of two groups in their circular variance (Oriana 4.1; Kovach Computing Services, Anglesey, UK). Amplitude and period length differences were calculated by two-tailed Student's t-tests.

## Author Contributions

P.Z. performed experiments, analyzed data and wrote the paper. R.A.R. performed experiments. C.M.P. performed experiments and analyzed data. G.E.D. and S.L. designed experiments, analyzed data, and wrote the paper.

## Supplementary Material

Supplementary Information SUPPLEMENTARY INFORMATION

Supplementary Information Supplementary Datafile 1

## Figures and Tables

**Table 1:** Amplitude of clock gene rhythms under control and alcohol diets

Gene name	Control Fold change Peak to Trough	Alcohol Fold change Peak to Trough	Ratio (Alcohol fed/control)
*clock*	**3.1**	**3.5**	**1.1**
*Bmal1*	**14.9**	**10.7**	**0.7**
*Npas2*	**38.5**	**8.0**	**0.2**
*per1*	**5.9**	**6.2**	**1.1**
*per2*	**6.2**	**3.3**	**0.5**
*per3*	**23.5**	**9.6**	**0.4**
*cry1*	**4.8**	**8.9**	**1.9**
*cry2*	**9.9**	**4.0**	**0.4**
*Rev-erbα*	**17.0**	**11.0**	**0.6**
*Rev-erbβ*	**6.9**	**2.1**	**0.3**
