# Supplementary material for: Disturbances in the murine hepatic circadian clock in alcohol-induced hepatic steatosis
Source: Sci Rep. 2014 Jan 16;4:3725. doi: 10.1038/srep03725 (PMC3893658; doi:10.1038/srep03725)

Disturbances in the murine hepatic circadian clock in alcohol-induced hepatic steatosis

Peng Zhou, Ruth A. Ross, Cameron M. Pywell, Giles E. Duffield, Suthat Liangpunsakul

SUPPLEMENTARY INFORMATION

Supplementary Information Figure Legends

Supplementary Datafile 1. Differentially expressed genes following chronic alcohol feeding treatment, with >2-fold and >1.5-<2.0-fold change filters. Peak phase value in circadian time [CT] provided where gene was identified as a clock gene or CCG. Rhythmic genes identified from Circa database of mouse liver 48-h circadian time course. Grey box, not rhythmic; black box, no information available on Circa database.

Supplementary Figure S1. Microarray and qRT-PCR analysis of liver following chronic alcohol treatment. (a) 166 and 206 genes from microarray experiment identified as differentially expressed (≥ 1.5 and < 2.0-fold changes compared to control treated mice) were up- and down-regulated, respectively. (b) Distribution of differentially expressed clock genes and CCGs (≥ 1.5 and < 2.0-fold change) arranged according to phase of peak expression. (c) Phase distribution of rhythmically expressed genes in alcohol fed mice found up- or down-regulated ≥ 1.5-fold and < 2.0 (black dots) or ≥ 2.0-fold (red dots). Data are log-transformed and plotted against their respective peak phase time (determined from Circa database). Differentially expressed clock genes and POGs (≥ 2.0-fold change) are highlighted with a number. (d) Correlation between microarray experiment (ZT2.5) and qRT-PCR gene expression fold-changes at ZT0 or ZT4, for differentially expressed clock genes and POGs (11 genes that are highlighted in Fig. 2D). R represents Pearson correlation coefficient value.

Supplementary Figure S2. CircWave cosinor analysis of liver gene expression in control and alcohol-fed mice across the 24-h diurnal cycle. In each graph, dots represent the normalized relative mRNA level of each animal from each time-specific group (vertical axis). Zeitgeber time (h) is on horizontal axis. The curve represents the best-fit fourier curve. The center of gravity (CG) is represented by the vertical bar. Mean of the entire data set is represented by the cross. Data sets that do not fit in a fourier curve are represented with ‡.

Supplementary Figure S1

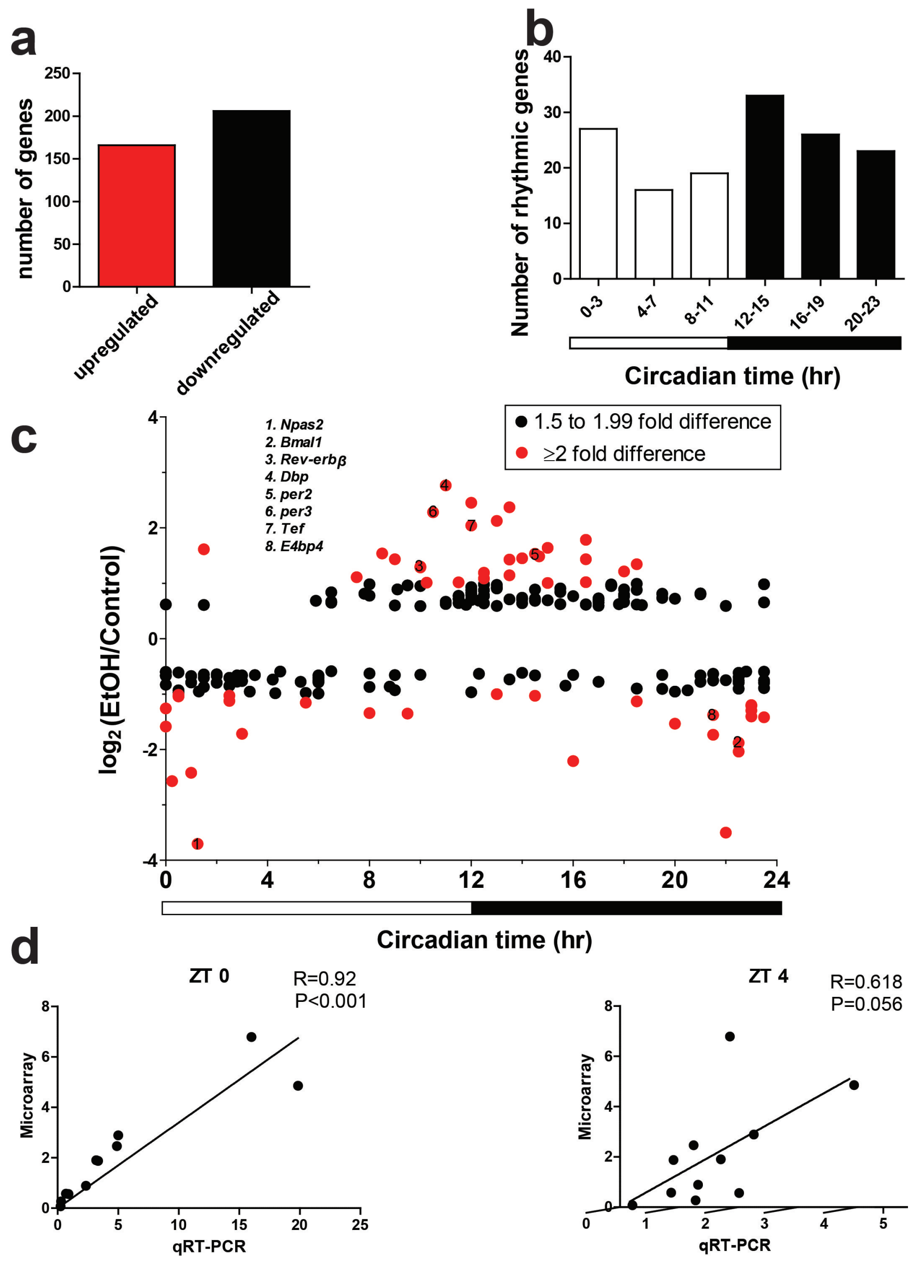

Supplementary Figure S2

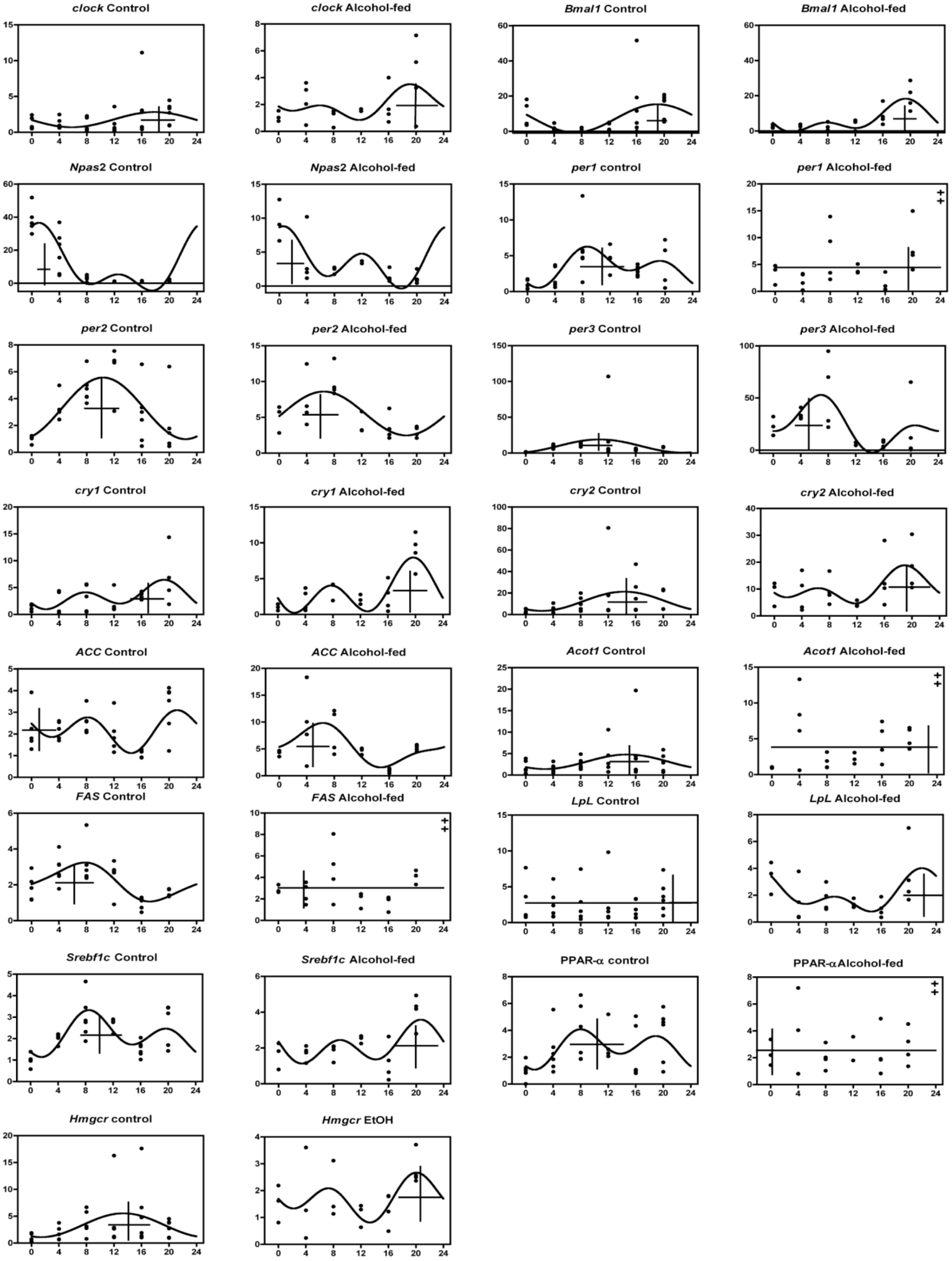

Supplement: Supplementary Information [file srep03725-s1.pdf]
